# Single‐cell and repertoire profiling reveals immune remodelling in paediatric upper airway: insights from adenoid hypertrophy

**DOI:** 10.1002/cti2.70101

**Published:** 2026-06-05

**Authors:** Chao Wang, Yufei Pan, Xiao Han, Kai Sun, Jing Li, Yuanyuan Lu, Zhenkun Yu

**Affiliations:** ^1^ Department of Otolaryngology‐Head and Neck Surgery, BenQ Medical Center The Affiliated BenQ Hospital of Nanjing Medical University Nanjing Jiangsu China; ^2^ Nanjing Medical Key Laboratory of Laryngopharynx and Head and Neck Neoplasm Nanjing Jiangsu China; ^3^ School of Medicine Southeast University Nanjing Jiangsu China

**Keywords:** adenoid hypertrophy, children, immune repertoire, obstructive sleep apnoea, single‐cell RNA sequencing

## Abstract

**Objectives:**

To characterise the immune cellular landscape and paired B‐cell and T‐cell receptor repertoires of hypertrophic adenoids in children with obstructive sleep apnoea.

**Methods:**

We performed 10x Genomics 5′ single‐cell RNA sequencing with paired V(D)J profiling on adenoid tissues from children (AH, *n* = 4; control, *n* = 4) and analysed 72 076 high‐quality cells.

**Results:**

Compared with controls, AH adenoids exhibited an expanded B‐cell compartment (62.26% vs 51.94%, *P* = 0.029) and a modest increase in plasmacytoid dendritic cells (0.37% vs 0.15%, *P* = 0.029), accompanied by reduced T‐cell representation (34.19% vs 44.54%, *P* = 0.029). Within the B‐cell compartment, GC B cell populations were expanded in AH, particularly Cycling B cells (15.07% vs 2.86%, *P* = 0.029), whereas memory B cells were reduced (21.48% vs 32.18%, *P* = 0.029). B‐cell receptor analysis demonstrated reduced somatic hypermutation frequencies across multiple mature B‐cell subsets in AH, accompanied by an isotype distribution skewed towards IgM with reduced class‐switched fractions. Although CD8^+^ tissue‐resident memory T‐cell (TRM) frequencies were similar between groups, CD8^+^ TRM in AH adenoids displayed interferon‐polarised and antigen‐processing signatures, and T‐cell clonal expansion occurred predominantly within this subset.

**Conclusion:**

These findings suggest that AH is not merely an anatomic cause of upper airway obstruction but also an immunologically active mucosal state associated with ongoing immune remodelling and antiviral defence.

## Graphical abstract

Single‐cell and paired V(D)J profiling show that paediatric adenoid hypertrophy features expanded proliferative germinal‐centre B cells with impaired maturation.

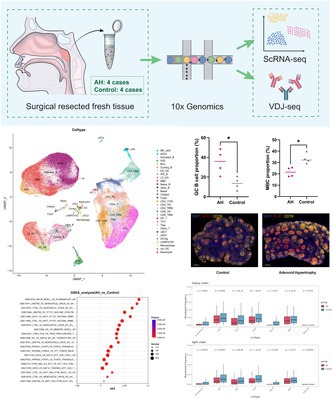

## Introduction

The adenoids, part of Waldeyer's ring—a collection of lymphoid tissue at the entrance of the upper aerodigestive tract—play a critical role in mucosal immune defence, particularly against airborne pathogens.^1^ In childhood, adenoids typically proliferate rapidly between ages 2 and 6, reaching maximal size around 6–8 years, followed by gradual involution during adolescence.^2^ Adenoid hypertrophy (AH) is the leading anatomical contributor to paediatric obstructive sleep apnoea (OSA), a disorder associated with neurocognitive, cardiovascular and behavioural sequelae.^3^, ^4^

Adenotonsillectomy (AT) is the primary intervention for paediatric OSA.^5^ While effective for relieving obstruction, surgery removes a mucosal immune organ during a critical developmental paediatric period. Increasing evidence suggests that immune activation may accompany AH rather than simple tissue enlargement. For example, B cells—the dominant lymphoid population in adenoids—may contribute to local humoral defence against viral and bacterial pathogens.^6^ Meanwhile, tissue‐resident memory T (TRM) cells, increasingly recognised as key mediators of rapid, site‐specific immune responses in barrier tissues, are thought to maintain long‐term local immune surveillance against recurrent respiratory pathogens.^7^, ^8^ However, the cellular composition, transcriptional states and antigen‐specific repertoires of immune cells in paediatric adenoids—particularly in hypertrophy—remain poorly characterised. Whether AH represents a purely pathological process or a transient state of heightened immune readiness is still unclear.

In this context, single‐cell transcriptomic approaches offer a powerful means to resolve disease‐associated immune heterogeneity by enabling unbiased, high‐resolution characterisation of cellular composition and functional states within complex tissues.^9^ This study aimed to investigate the cellular and transcriptional heterogeneity underlying AH in children, utilising single‐cell RNA sequencing and immune repertoire analysis. Specifically, we seek to delineate alterations in cellular composition and functional states within hypertrophic adenoids, with a particular focus on TRM cells, to advance understanding of both the pathogenesis of AH and its mucosal immune defence functions.

## Results

### Cohort description

Eight children were enrolled (AH, *n* = 4; control, *n* = 4). As shown in Table 1, the two groups were comparable in age, sex distribution and body mass index. White blood cell count (difference, −0.14; 95% CI, −2.60 to 2.86) and C‐reactive protein level (difference, −0.10 mg/L; 95% CI, −3.50 to 3.90 mg/L) did not differ significantly between groups. Tonsillar size (grade III–IV) was observed in 75% of children with AH and in all controls (difference, 0.25; 95% CI, −0.40 to 0.78). PSQ total score (difference, 0.16; 95% CI, −0.23 to 0.55) and OSA‐18 scores (difference, 4.0; 95% CI, −3.0 to 11.0), as well as polysomnographic parameters, including OAHI (difference, 6.1; 95% CI, −1.0 to 14.1) and oxygen nadir (82% vs 89%; difference, −9%; 95% CI, −19% to 1%), did not differ significantly between groups. Taken together, the 2 groups showed no major baseline imbalances within this small cohort.

**Table 1:** Baseline patient demographic characteristics

Characteristic	Patients, no. (%)		
	AH	Control	Difference (95% CI)
Age, median (IQR), years	6.50 (5.25, 8.50)	8.50 (5.75, 9.00)	−1.50 (−4.00 to 2.00)^a^
Sex			
Male	2 (50%)	1 (25%)	0.25 (−0.41 to 0.72)^b^
Female	2 (50%)	3 (75%)	
BMI, kg/m^2^	20.30 (18.91, 20.83)	17.99 (16.43, 20.15)	1.93 (−0.74 to 4.57)^a^
Tonsillar size III and IV	3 (75%)	4 (100%)	0.25 (−0.40 to 0.78)^b^
Allergy history	2 (50%)	2 (50%)	0 (0.0 to 0.0)^b^
Smoke exposure	0 (0%)	0 (0%)	0 (0.0 to 0.0)^b^
PSQ total score, median (IQR)	0.45 (0.39, 0.69)	0.27 (0.24, 0.51)	0.16 (−0.23 to 0.55)^a^
OSA‐18 total score, median (IQR)	61.5 (59.25, 77.25)	59.5 (55.25, 63.75)	4.0 (−3.0 to 11.0)^a^
White blood cell count, ×10^9^/L	6.80 (5.68, 7.97)	7.42 (6.35, 8.15)	−0.14 (95% CI, −2.60 to 2.86)^a^
C‐reactive protein, mg/L	2.10 (1.75, 3.03)	2.50 (1.90, 3.38)	−0.10 (95% CI, −3.50 to 3.90)^a^
OAHI, median (IQR)	12.00 (8.10, 17.10)	3.90 (2.88, 7.93)	6.10 (−1.00 to 14.1)^a^
Oxygen nadir, median (IQR), %	82% (74%, 84%)	89% (84%, 96%)	−9% (−19% to 1%)^a^

BMI, body mass index; OAHI, Obstructive Apnea–Hypopnea Index; OSA, Obstructive Sleep Apnea.

^a^ Median difference.

^b^ Percentage difference.

### Construction of a paediatric adenoid single‐cell atlas

**Figure 1:**
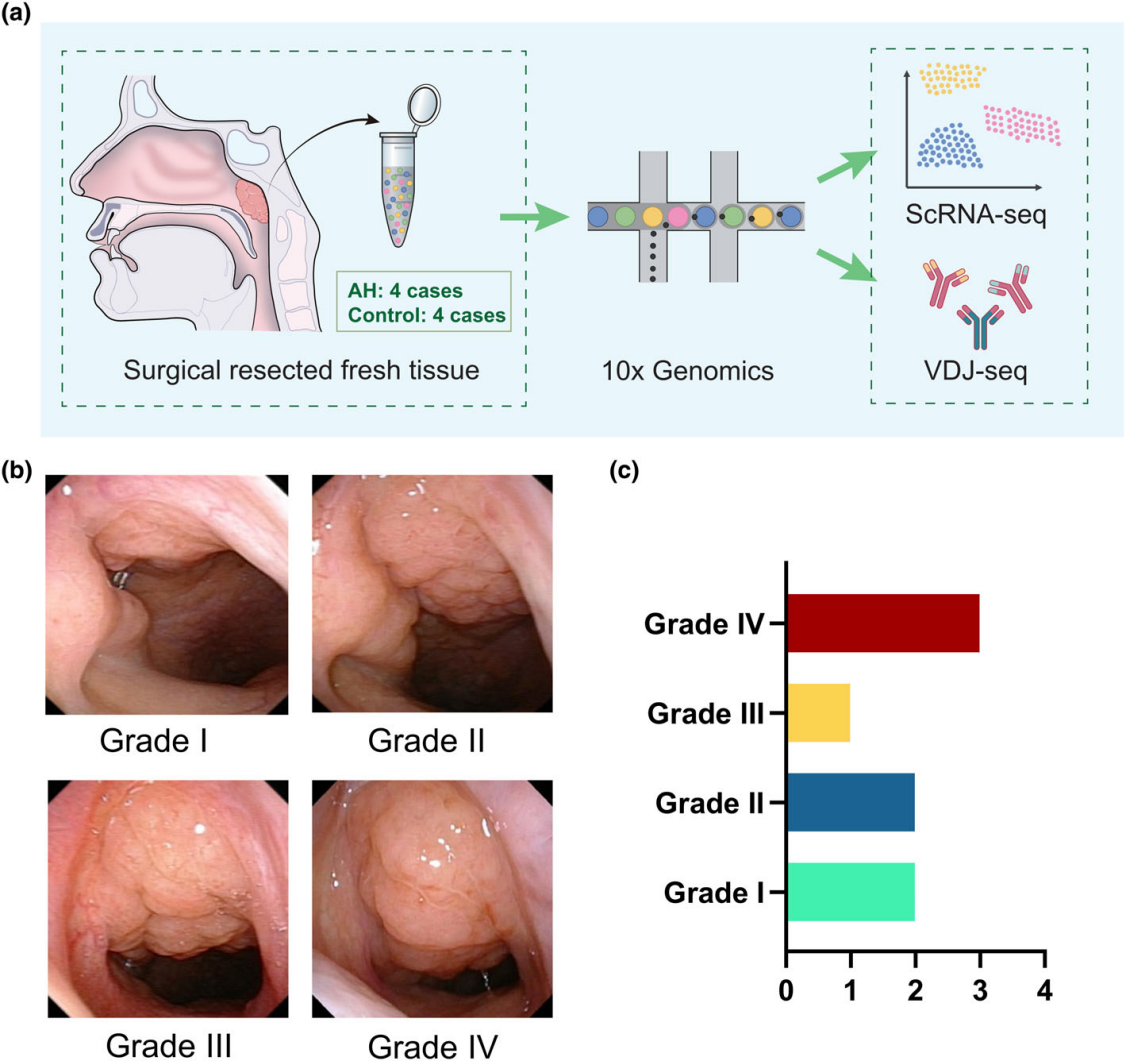
**(a)** Intraoperative removal of adenoid tissue from a paediatric patient, followed by immediate processing for single‐cell RNA sequencing and paired V(D)J repertoire profiling. **(b)** Schematic representation of adenoid hypertrophy graded from I to IV according to the percentage of choanal obstruction on nasopharyngoscopy. **(c)** Distribution of adenoid hypertrophy grades among specimens included in the present study.

**Figure 2:**
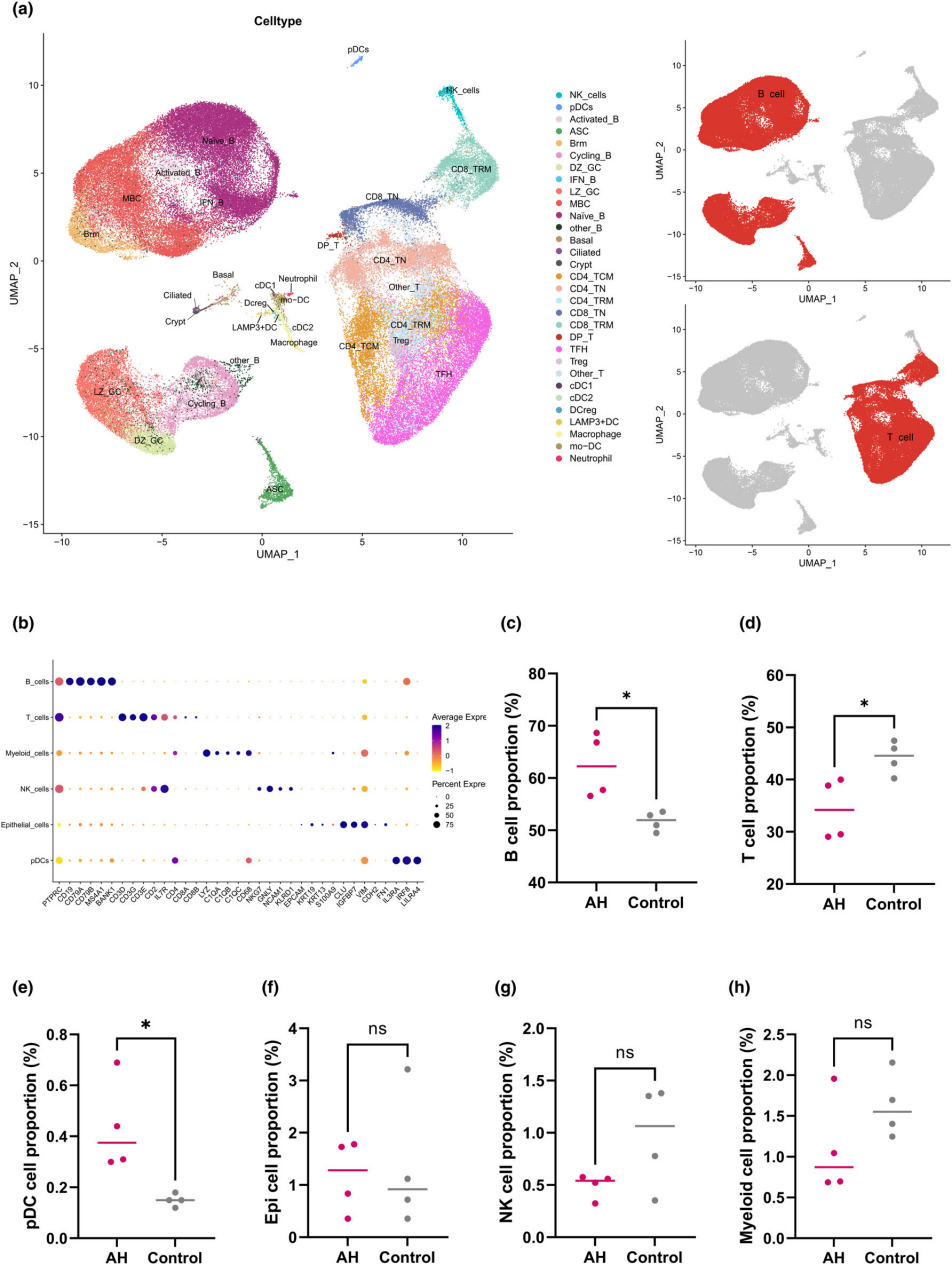
(**a**) UMAP plot showing all single cells from adenoid specimens, coloured by unsupervised clustering. (**b**) Major cell types annotated based on canonical marker gene expression. Comparison of B cell (**c**), T cell (**d**), plasmacytoid dendritic cells (**e**), epithelial cell (**f**), natural killer (NK) cell (**g**), and myeloid cell (**h**) proportions between the AH and control groups. *, *P* < 0.05; ns, not significant.

Fresh adenoid tissue from each participant was processed on the 10× Genomics Chromium platform for paired single‐cell RNA‐seq and V(D)J repertoire profiling (Figure 1a). After stringent quality control—removing doublets, low‐complexity barcodes and cells with excessive mitochondrial transcripts—we retained 72 076 high‐quality single‐cell transcriptomes across eight samples. Library complexity and sequencing performance were robust: the median genes per cell were 2005 (IQR 1952–2229); the mean reads per cell averaged 43 109 (median 39 422; IQR 31675–52 515; per‐sample range 30 168–64 194); and sequencing saturation had a median of 70.8% (IQR 63.9–73.3%; range 59.8–78.6%). Unsupervised integration of all samples revealed six canonical immune and structural lineages (Figure 2a): B cells, T cells, myeloid cells (monocytes/macrophages), NK cells, plasmacytoid dendritic cells (pDCs) and epithelial cells. Lineages were validated by canonical marker expression (Figure 2b). B cells expressed CD19, CD79A and MS4A1; T cells expressed CD3D, CD3E and CD2; myeloid cells were characterised by LYZ, FCER1A and C1QC; NK cells expressed NKG7, GNLY and KLRD1; epithelial cells expressed EPCAM, KRT19 and KRT5; and pDCs were identified by IL3RA, LILRA4 and FCER1A. Compared with Grade I–II adenoids (Figure 2c–h), AH samples showed higher relative B‐cell proportions (median, 62.26%; IQR, 57.43–67.28% vs 51.94%; 50.62–53.05%; *P* = 0.029) and higher relative pDC representation (0.37%; 0.31–0.50% vs 0.15%; 0.14–0.16%; *P* = 0.029), accompanied by lower relative T‐cell proportions (34.19%; 29.40–39.14% vs 44.54%; 42.39–46.33%; *P* = 0.029). NK cells (0.54%; 0.47–0.56% vs 1.06%; 0.67–1.36%; *P* = 0.20), myeloid cells (0.87%; 0.69–1.27% vs 1.55%; 1.36–1.81%; *P* = 0.20) and epithelial cells (1.28%; 0.72–1.74% vs 0.92%; 0.63–1.64%; *P* = 0.89) did not differ significantly between groups. Collectively, these data suggest compositional remodelling in AH, with relative expansion of the B‐cell compartment, modest enrichment of pDCs and lower T‐cell representation.

### Germinal‐centre hyperactivity and proliferative bias in the B‐cell compartment

**Figure 3:**
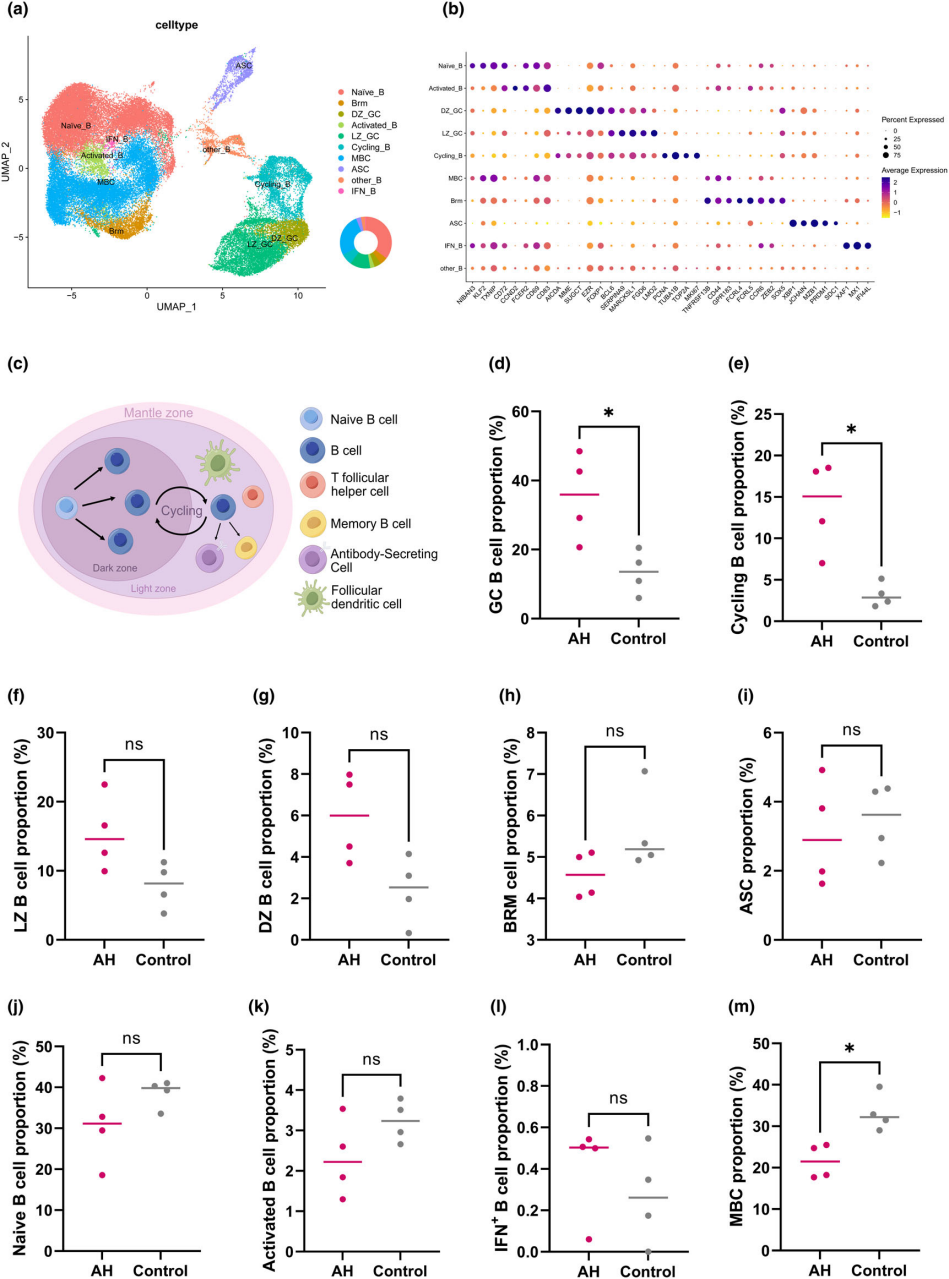
**(a)** Uniform manifold approximation and projection (UMAP) plot of B‐cell subclusters. **(b)** Dot plot of canonical marker genes across B‐cell subclusters. **(c)** Schematic illustration of B‐cell maturation within the germinal centre (GC), from naïve B cells through germinal centre reactions to fully differentiated memory or plasma cells. **(d–m)** Comparison of B‐cell subset proportions between the adenoid hypertrophy (AH) and control groups, including GC_B **(d)**, Cycling_B **(e)**, LZ_B **(f)**, DZ_B **(g)**, BRM **(h)**, ASC **(i)**, Naïve_B **(j)**, Activated_B **(k)**, IFN_B **(l)**, MBC **(m)**. *, *P* < 0.05; ns, not significant.

**Figure 4:**
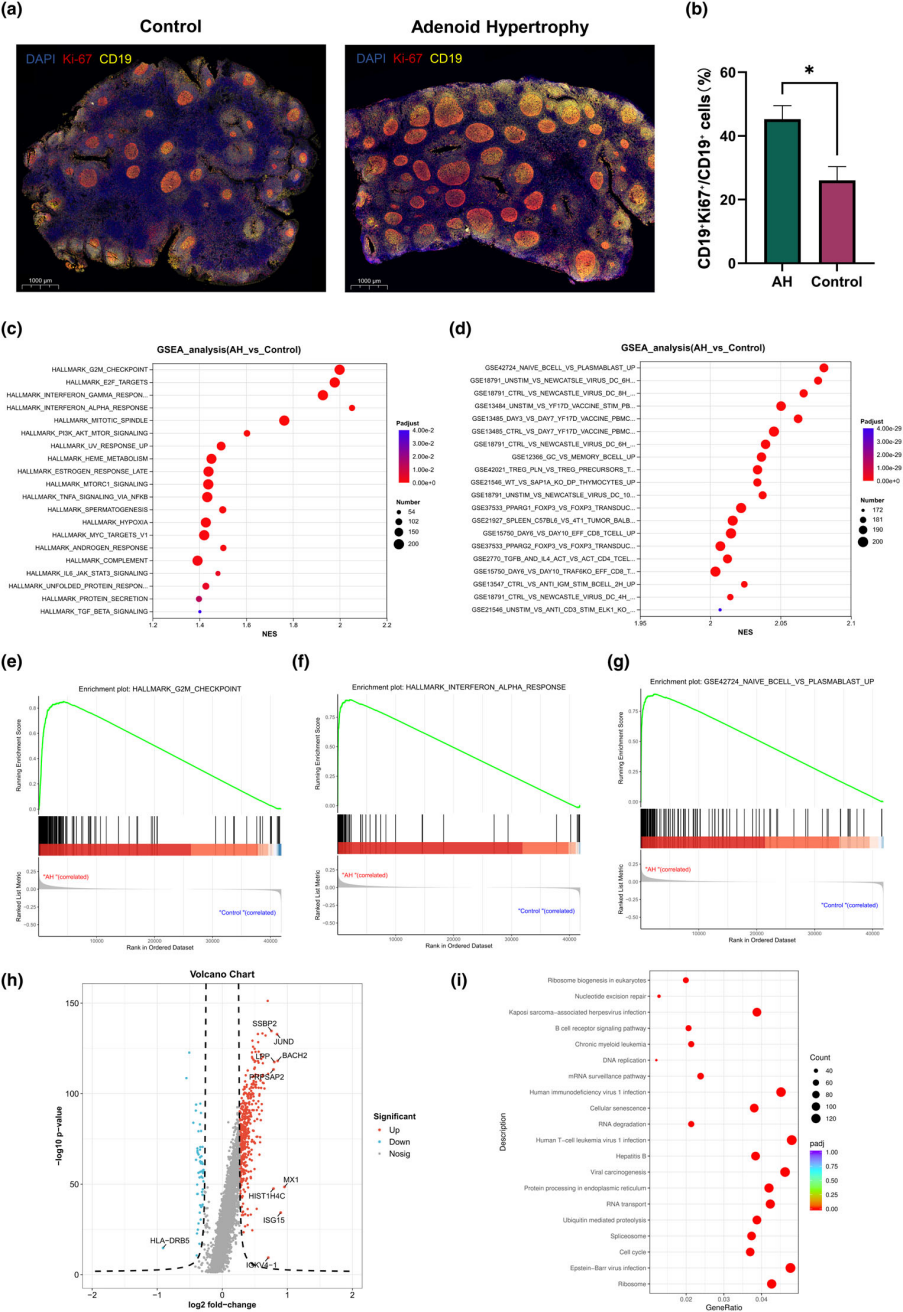
**(a)** Representative immunofluorescence staining of adenoid tissue showing CD19^+^Ki67^+^ proliferating B cells in the adenoid hypertrophy (AH) and control groups. **(b)** Quantification of the proportion of CD19^+^Ki67^+^ cells among CD19^+^ cells in germinal centres, performed using QuPath, with statistical comparison between the AH and control groups. *, *P* < 0.05. **(c, d)** Gene Set Enrichment Analysis (GSEA) plots displaying the global enrichment of HALLMARK and immunologic signatures in AH B cells. Full GSEA results for the HALLMARK and C7 immunologic signature collections are provided in Supplementary tables 1 and 2. **(e–g)** Individual enrichment curves highlighting activated G2M checkpoint, interferon response and plasmablast differentiation programmes. **(h)** Volcano plot illustrating differential gene expression in Cycling B cells (threshold: |log_2_FC| ≥ 1.5 and FDR < 0.05). **(i)** KEGG pathway enrichment analysis of Cycling B cells.

Re‐clustering of B cells identified nine transcriptionally distinct subpopulations (Figure 3a): Naïve_B, BRM, dark‐zone germinal centre (DZ_GC), light‐zone germinal centre (LZ_GC), Activated_B, Cycling_B, memory B cells (MBC), antibody‐secreting cells (ASC) and IFN‐responsive B cells (IFN_B). These annotations were supported by canonical marker expression, as shown in Figure 3b. These subclusters collectively recapitulated the canonical B‐cell maturation continuum from naïve B cells through GC reactions towards memory and antibody‐secreting fates (Figure 3c). Cycling_B cells showed higher relative proportions in AH samples (median, 15.07%; IQR, 9.54–18.30%) compared with controls (2.86%; 2.10–4.24%; *P* = 0.029), whereas MBC were relatively reduced in AH (21.48%; 17.97–25.09%) versus controls (32.18%; 30.24–36.21%; *P* = 0.029). DZ_GC (6.00%; 4.10–7.73% vs 2.53%; 1.15–3.62%) and LZ_GC (14.59%; 11.28–19.52% vs 8.17%; 5.18–10.52%) showed higher proportions in AH with borderline significance (both *P* = 0.057). Naïve_B, BRM, Activated_B, ASC and IFN_B did not differ significantly between groups (Figure 3f–l). Quantitative immunofluorescence analysis using QuPath showed that the proportion of CD19^+^Ki67^+^ cells among GC CD19^+^ cells was higher in the AH group than in controls (Figure 4b; *P* = 0.029). Collectively, these results indicate enhanced GC and proliferative B‐cell activity with relative depletion of memory B cells in AH adenoids.

Consistent with these compositional changes, GSEA of the overall B‐cell compartment demonstrated global enrichment of proliferative and immune‐activation programmes in AH relative to controls (Figure 4c and d). Representative enrichment plots highlighted activation of the G2M checkpoint, interferon response and plasmablast differentiation signatures (Figure 4e–g), further supporting a state of heightened GC activity and immune remodelling in AH. Differential expression analysis (Figure 4h) identified 7030 genes in Cycling_B cells (AH vs control; FDR‐adjusted *P* < 0.05), including 4907 up‐regulated and 2123 down‐regulated genes. Up‐regulated transcripts highlighted proliferative machinery—MKI67, TOP2A, PCNA, CCNB1, CDK1, AURKA/AURKB and UBE2C—as well as chromatin/translation‐related genes (eg, HIST1H4C) and regulators (BACH2, JUND). KEGG over‐representation confirmed strong enrichment of biosynthetic and cell‐cycle programmes: Ribosome (120/2810 genes, *P* = 1.9 × 10^−31^), spliceosome (105/2810, *P* = 3.4 × 10^−20^), cell cycle (104/2810, *P* = 2.5 × 10^−20^), ubiquitin mediated proteolysis (109/2810, *P* = 2.3 × 10^−19^) and proteasome (30/2810, *P* = 8.5 × 10^−4^) (Figure 4i). Virus‐related modules were also enriched, including Epstein–Barr virus infection (134/2810, *P* = 1.6 × 10^−20^), Hepatitis C (89/2810, *P* = 2.0 × 10^−7^), measles (81/2810, *P* = 2.8 × 10^−7^), influenza A (90/2810, *P* = 1.3 × 10^−6^) and human cytomegalovirus infection (114/2810, *P* = 6.2 × 10^−5^). These virus terms shared a leading‐edge set of interferon/antiviral genes present among Cycling_B DEGs—ISG15, MX1, OAS1/OAS2, STAT1/STAT2, IRF7/IRF9—indicating that Cycling_B cells in AH adenoids concurrently engage proliferative and antiviral transcriptional programmes.

### BCR repertoire analysis: SHM and CSR

**Figure 5:**
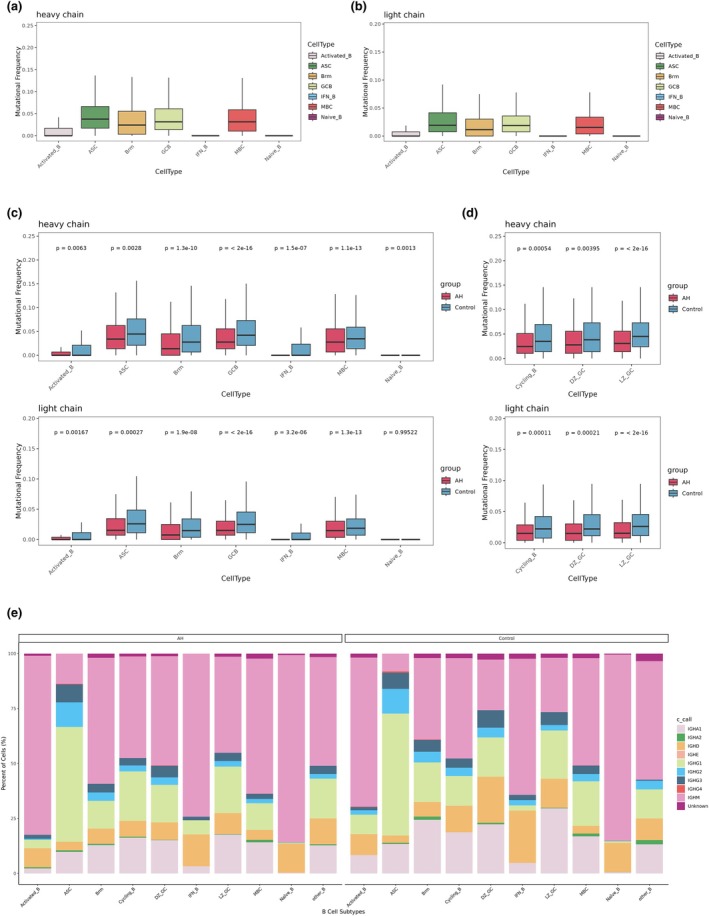
**(a)** Heavy‐chain somatic hypermutation (SHM) frequencies across B‐cell subsets. **(b)** Light‐chain somatic hypermutation (SHM) frequencies across B‐cell subsets. **(c)** Comparison of SHM frequencies (including heavy chain and light chain) between adenoid hypertrophy (AH) and control groups across B‐cell subsets. **(d)** Comparison of SHM frequencies (including heavy chain and light chain) between AH and control groups across germinal centre (GC) B‐cell subsets. **(e)** Class‐switch recombination (CSR) analysis showing immunoglobulin isotype distribution across B‐cell subsets in AH and control groups.

To characterise the extent of affinity maturation within the hypertrophic microenvironment, we quantified SHM frequencies in both immunoglobulin heavy and light‐chain sequences (Figure 5a–d). Despite the marked expansion of GC B cells observed in adenoid hypertrophy (AH), the mutational load was significantly attenuated across nearly all mature B‐cell subsets compared with the control group. In the heavy chain repertoire, AH specimens exhibited significantly lower SHM frequencies in GC B cells (*P* < 0.001), MBC (*P* < 0.001), BRM cells (*P* < 0.001) and IFN‐responsive B cells (*P* < 0.001). Mutational attenuation was also evident in ASC cells (*P* = 0.003) and activated B cells (*P* = 0.006). This trend was consistently mirrored in the light‐chain repertoire, with the most pronounced reductions identified in GC B (*P* < 0.001), MBC (*P* < 0.001) and BRM (*P* < 0.001). Notably, while a statistically significant difference was observed in the heavy chain of naive B cells (*P* = 0.001), light‐chain mutation frequencies remained comparable between groups (*P* = 0.995), consistent with their germline status. Collectively, these data suggest that while AH is characterised by robust B‐cell proliferation, these cells harbour a qualitatively less mature mutational landscape.

To characterise the structural diversity of the B‐cell receptor repertoire, we profiled the usage of immunoglobulin heavy chain variable (IGHV) and joining (IGHJ) gene segments, as well as the length distribution of complementarity‐determining region 3 (CDR3) (Supplementary figure 1A–C, G). Following this, CSR analysis demonstrated distinct immunoglobulin isotype distributions between groups (Figure 5e). Within the GC compartment (DZ_GC, LZ_GC and Cycling_B), AH showed higher IgM and lower class‐switched fractions in DZ and LZ. In DZ_GC, IgM was 49.7% vs 23.0%, with IgD 7.9% vs 21.0% and IgA1 15.1% vs 22.3%. In LZ_GC, IgM was 43.7% vs 24.7% and IgA1 17.6% vs 29.6%. In Cycling_B, IgG1 was higher in AH (22.5% vs 13.5%). Outside the GC, MBC showed IgM 61.4% vs 48.8% and IgG1 12.1% vs 20.3%. Activated_B showed IgA1 2.3% vs 8.4%, IgG1 3.8% vs 8.9% and IgM 81.4% vs 67.8%. BRM showed IgA1 12.9% vs 24.4%, IgG1 12.6% vs 18.0% and IgM 57.3% vs 37.0%. IFN_B showed IgD 14.5% vs 23.8% and IgM 74.2% vs 61.9%. In ASC, IgM was 13.7% vs 8.1%. Overall, apart from Cycling_B, most subsets showed higher IgM in AH and higher IgA1/IgG1 in controls.

### Transcriptional heterogeneity and subset distribution of T cells

**Figure 6:**
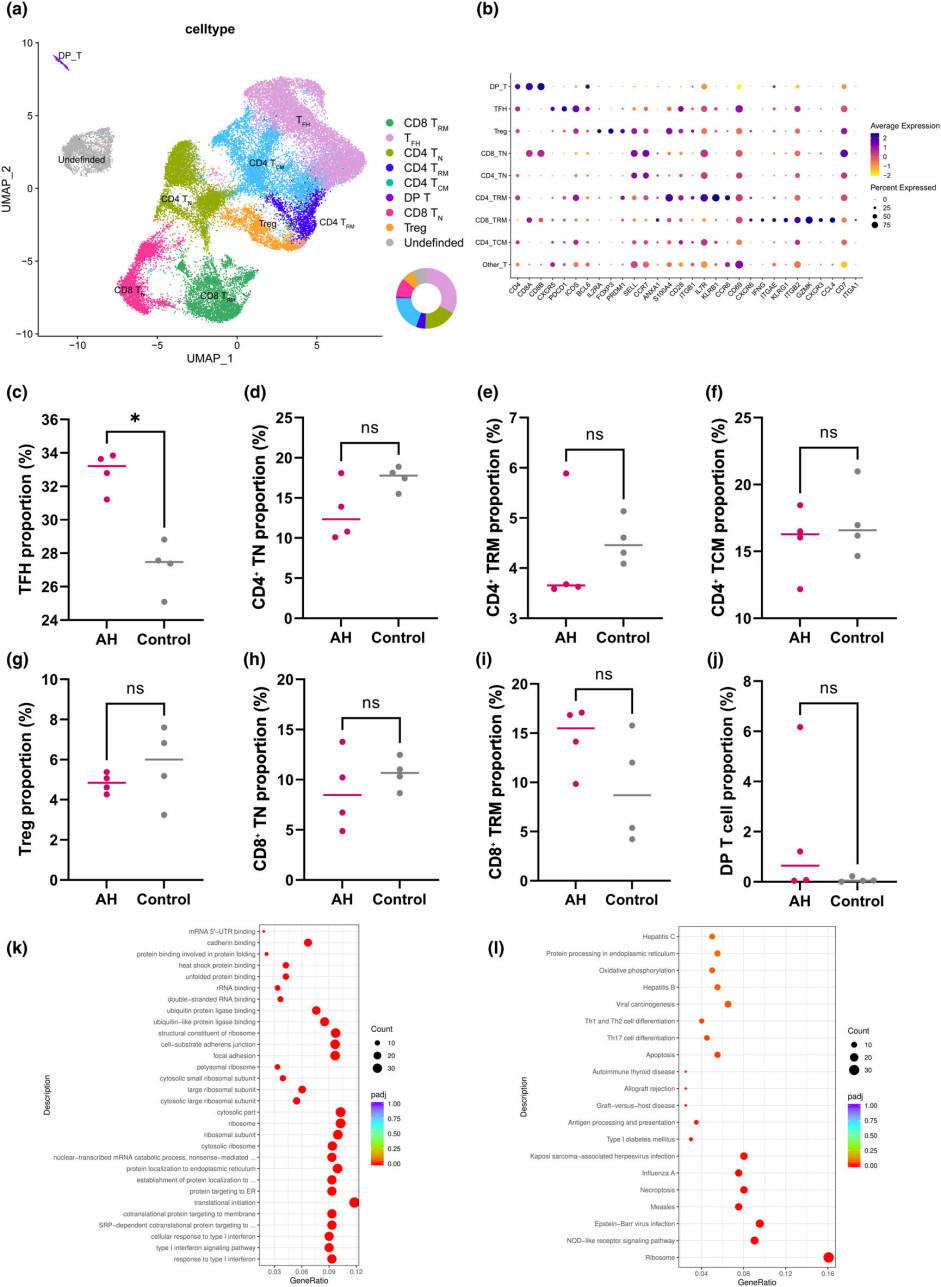
**(a)** Uniform manifold approximation and projection (UMAP) plot of T‐cell subclusters from all adenoid samples. **(b)** Dot plot of canonical marker genes across T‐cell subclusters. **(c–j)** Comparison of T‐cell subset proportions between the adenoid hypertrophy (AH) and control groups, including TFH **(c)**, CD4^+^ TN **(d)**, CD4^+^ TRM **(e)**, CD4^+^ TCM **(f)**, Treg **(g)**, CD8^+^ TN **(h)**, CD8^+^ TRM **(i)** and DP_T **(j)**. *, *P* < 0.05; ns, not significant. **(k)** GO enrichment analysis of CD8^+^ TRM subsets. **(l)** KEGG pathway enrichment analysis of CD8^+^ TRM subsets.

Re‐clustering of T cells resolved nine transcriptionally distinct subsets (Figure 6a): CD8^+^ TRM, T follicular helper (TFH) cells, naïve and central memory CD4^+^ T cells (CD4^+^ TN, CD4^+^TCM), CD4^+^ tissue‐resident memory T cells (CD4^+^ TRM), double‐positive T cells (DP_T), naïve CD8^+^ T cells (CD8^+^ TN), regulatory T cells (Treg) and an undefined population. These annotations were supported by canonical marker expression, as shown in Figure 6b. As shown in Figure 6c, TFH cells exhibited higher relative proportions in AH samples (median, 33.21%; IQR, 32.01–33.74%) compared with controls (27.47%; IQR, 26.24–28.19%; *P* = 0.029). The undefined population was more abundant in controls (7.99%; 6.93–8.65%) than in AH (3.16%; 1.98–5.66%), showing a near‐significant trend (*P* = 0.057). DP_T cells tended to be increased in AH (0.64%; 0.06–3.69%) versus controls (0.05%; 0.02–0.14%; *P* = 0.20). The remaining subsets, including CD8^+^ TRM, CD4^+^ TN, CD8^+^TN, Treg, CD4^+^ TRM and CD4^+^ TCM, showed no statistically significant differences between groups (Figure 6d–j).

Despite comparable CD8^+^ TRM frequencies across groups, differential expression (AH vs control) identified 346 transcripts (FDR < 0.05; 193 up‐regulated, 153 down‐regulated). Up‐regulated genes marked a type I interferon/antiviral and antigen‐presentation programme, including ISG15, IFI6, MX1/MX2, IRF7, STAT1/STAT2, OAS1/OAS2, EIF2AK2 (PKR), DDX58 (RIG‐I), EPSTI1, LY6E, HERC5, TRIM22, GBP1/2/4/5 and BST2, with increased HLA‐B/HLA‐C and TAP1/TAP2 and higher CD38, LAG3 and TNFSF10. Down‐regulated genes involved trafficking/tonic signalling (eg, CXCR4, CXCR6, IL7R and FOXP1), and cytotoxic signatures showed mixed directionality (PRF1 up; CTSW and GZMM down). GO enrichment analysis (Figure 6k) corroborated these patterns, highlighting response to type I interferon (30/322; 1.13 × 10^−25^), type I interferon signalling pathway (29/322; 2.02 × 10^−25^), cellular response to type I interferon (29/322; 2.02 × 10^−25^), response to virus (39/322; 6.80 × 10^−18^), defence response to virus (34/322; 7.21 × 10^−18^), negative regulation of viral genome replication (13/322; FDR = 1.89 × 10^−8^), regulation of apoptotic signalling pathway (22/322; 9.84 × 10^−4^), antigen processing and presentation (13/322; 1.68 × 10^−2^), and antigen processing and presentation of peptide antigen (11/322; 2.75 × 10^−2^). KEGG (Figure 6l) over‐representation corroborated these patterns, highlighting ribosome (32/199; FDR = 7.30 × 10^−19^), NOD‐like receptor signalling (18/199; 4.45 × 10^−5^), Epstein–Barr virus infection (19/199; 4.74 × 10^−5^), measles (15/199; 1.39 × 10^−4^), necroptosis (16/199; 1.40 × 10^−4^) and influenza A (15/199; 6.31 × 10^−4^), antigen processing and presentation (7/199; 7.22 × 10^−3^), graft‐versus‐host disease, allograft rejection, autoimmune thyroid disease, apoptosis, Th17 cell differentiation, Th1/Th2 differentiation and viral carcinogenesis (all FDR < 0.05). Collectively, AH‐derived CD8^+^ TRM exhibit an interferon‐polarised, antigen‐processing‐competent state with concurrent up‐regulation of inhibitory checkpoints and cell‐death pathways.

### TCR repertoire and antigen annotation

**Figure 7:**
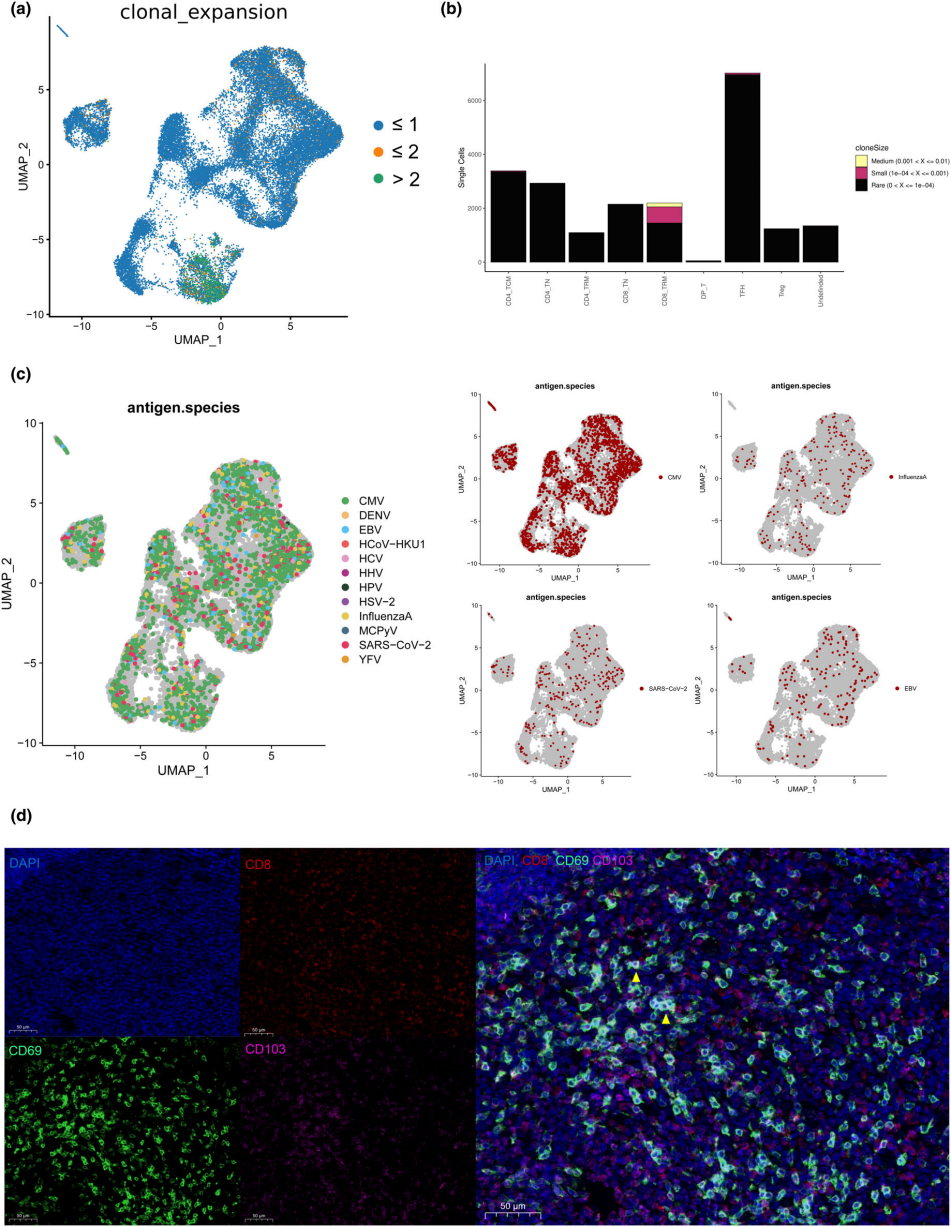
**(a)** Distribution of clonal expansion within T cells, with the majority of expanded clones localised in the CD8^+^ TRM subset. **(b)** Comparison of T‐cell clonal size among T‐cell subsets. **(c)** Antigen annotation of the T‐cell receptor (TCR) clonotypes, with top 4 specificities for cytomegalovirus (CMV), influenza A, SARS‐CoV‐2 and Epstein–Barr virus (EBV). **(d)** Representative immunofluorescence staining showing CD8^+^ TRM cells in adenoid tissue.

We first examined the global TCR landscape by analysing V‐J gene pairing and CDR3 length distributions, which revealed the baseline complexity of the T‐cell compartment (Supplementary figure 1D–F, I). Subsequent analysis of clonal dynamics showed that the clonal expansion of T cells occurred predominantly within CD8^+^ tissue‐resident memory T cells (Figure 7a and b). Antigen annotation using the VDJdb pipeline ranked mapped public clonotypes by their relative abundance in annotated TCRs (Figure 7c), with the highest representation directed against CMV (*n* = 1898), SARS‐CoV‐2 (*n* = 266), influenza A (*n* = 219) and EBV (*n* = 173). The presence of CD8^+^ tissue‐resident memory T (TRM) cells in adenoid tissue was further supported by immunofluorescence staining (Figure 7d).

### Altered myeloid cells landscape and functional pathways

**Figure 8:**
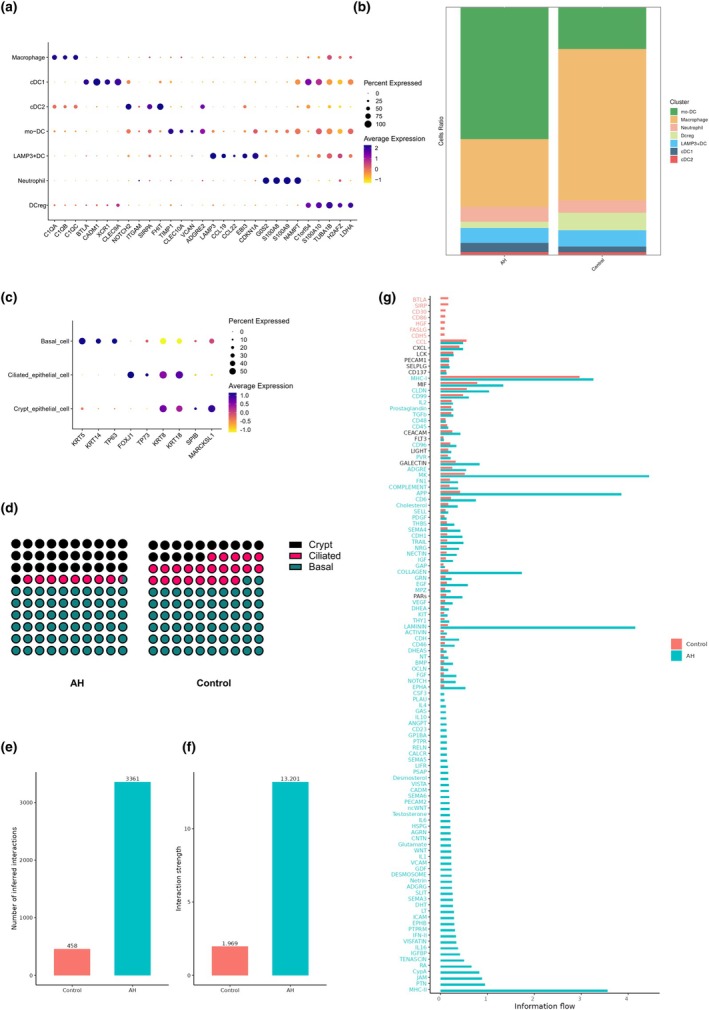
**(a)** Dot plot of canonical marker genes across myeloid cell subclusters. **(b)** Proportions of myeloid cell subclusters in adenoid hypertrophy and control groups. **(c)** Dot plot of canonical marker genes across epithelial cell subclusters. **(d)** Proportional distribution of crypt, ciliated and basal epithelial cells in the adenoid hypertrophy (AH) and control groups. In the AH group, crypt epithelial cells account for 30.77% of the total epithelial cells, basal cells for 60.36% and ciliated epithelial cells for 8.88%. In the control group, crypt epithelial cells make up 14.81%, basal cells comprise 62.04%, and ciliated epithelial cells represent 23.15%. **(e, f)** Cell–cell communication in AH and control groups. Bar plots show the number of inferred interactions **(e)** and interaction strength **(f)**. **(g)** Diagram of information flow among epithelial subclusters and CD8^+^ TRM cells.

Re‐clustering of myeloid cells identified seven transcriptionally distinct subsets (Figure 8a): monocyte‐derived dendritic cells (mo‐DC), macrophages, neutrophils, regulatory dendritic cells (DCreg), LAMP3^+^ dendritic cells (LAMP3^+^DC), type 1 conventional DCs (cDC1) and type 2 conventional DCs (cDC2). mo‐DCs demonstrated higher relative representation in AH samples (median, 59.75%; IQR, 56.97–60.89%) compared with controls (18.31%; 13.05–22.92%; *P* = 0.029), whereas macrophages were reduced in AH (24.52%; 22.25–29.22%) versus controls (62.79%; 58.15–65.77%; *P* = 0.029). The remaining subsets—including DCreg, neutrophils, LAMP3^+^DC, cDC1 and cDC2—did not differ significantly between groups. Collectively, these findings indicate a myeloid compartmental shift in AH characterised by relative enrichment of mo‐DCs and depletion of macrophages.

### Epithelial compositional shifts altered cellular crosstalk

Three epithelial subtypes were identified (Figure 8c): crypt epithelial cells, basal cells and ciliated epithelial cells. Given the small epithelial yield (each group < 200 epithelial cells), group‐level proportions without inferential testing are reported. In AH adenoids, basal cells comprised 60.36% of epithelial cells, crypt epithelial cells 30.77% and ciliated epithelial cells 8.88%; the corresponding values in controls were 62.04%, 14.81% and 23.15%, respectively (Figure 8d). These exploratory data suggest relative enrichment of crypt epithelium and reduction of ciliated epithelium in AH, with broadly comparable basal‐cell fractions between groups.

To investigate whether CD8^+^ TRM cells actively shape the epithelial microenvironment in AH, we performed intercellular communication analysis focused on ligand–receptor signalling between CD8^+^ TRM and epithelial cells. The total number of inferred interactions between these two populations was markedly increased in AH compared to controls (3316 vs 458; Figure 8e), accompanied by a fourfold increase in overall interaction strength (13.201 vs 1.969; Figure 8f). Pathway‐level comparison further revealed widespread enhancement of pro‐inflammatory and immune‐modulating signals in AH (Figure 8g). Notably enriched pathways included BAFF, CD40, IL6, CXCL, CCL, IL10, TGFβ, OSM, VEGF and LIGHT, all known to regulate epithelial integrity, immune activation or local inflammation. Specific ligand–receptor pairs contributing to these signals included TGFB1–TGFBR1/2, CD40L–CD40 and CXCL10–CXCR3. In contrast, homeostatic pathways, such as PTN, GAS and PSAP showed reduced communication in AH. These exploratory data indicate that CD8^+^ TRM cells in AH adenoids exhibit enhanced paracrine signalling towards epithelial cells, characterised by an inflammatory and remodelling‐oriented profile. This shift may contribute to sustained epithelial activation, barrier dysfunction and chronic tissue remodelling observed in AH.

## Discussion

### The ‘quantity vs quality’ paradox in B‐cell maturation

To our knowledge, this is the first study to compare hypertrophic and physiologic‐sized adenoids using 5′ single‐cell RNA sequencing (scRNA‐seq) integrated with paired V(D)J immune repertoire profiling. Our findings reveal a fundamental ‘quantity versus quality’ paradox in the immune landscape of paediatric AH. Although AH was characterised by marked expansion of GC B cells, these populations exhibited significantly lower SHM frequencies across both heavy and light chains than controls. Parallel to this mutational attenuation, we observed an immunoglobulin isotype distribution skewed towards IgM, with reduced class‐switched fractions, particularly IgG1 and IgA1. These findings provide functional support for the transcriptional ‘differentiation blockade’ recently described by Kang *et al*.^10^ who identified a PAX5‐associated defect impairing the transition from naïve to more differentiated B‐cell states in severe AH. Extending that model, our results suggest that B cells in AH are able to enter the GC programme and proliferate, yet fail to complete effective affinity maturation and isotype diversification. This interpretation is further supported by our GSEA, which showed persistent enrichment of a naïve‐like transcriptional signature in the overall B‐cell compartment.

The concurrent reduction in SHM, CSR and memory B‐cell output suggests that the GC response in AH is quantitatively expanded but qualitatively inefficient. In physiologic GC reactions, SHM and CSR are coupled to affinity‐based selection and the generation of long‐lived MBCs.^11^, ^12^ However, emerging evidence indicates that CSR is initiated largely before full GC maturation and becomes relatively infrequent within established germinal centres, making IgM‐dominated GCs biologically plausible rather than contradictory.^13^ Thus, the increased IgM fraction observed in AH should not be interpreted as absence of GC activity; instead, it may indicate impaired class‐switch entry or premature arrest of maturation before effective GC export. Moreover, chronic hypoxic and inflammatory stress within hypertrophic adenoids may further constrain AID‐dependent CSR and qualitative B‐cell maturation. At the same time, the IgM‐enriched profile may also reflect a mucosa‐adapted, first‐line humoral defence strategy in a tissue continuously exposed to inhaled antigens. In this setting, hypertrophic adenoids appear to favor sustained proliferative activation over efficient differentiation into durable, high‐affinity memory compartments.

This interpretation is also consistent with the distinctive biology of upper‐airway mucosa‐associated lymphoid tissue. Adenoids and tonsils are specialised inductive sites for continuous environmental antigen sampling, and their B‐cell responses are shaped by local mucosal demands rather than systemic humoral priorities alone.^6^ Prior studies have shown that these tissues are rich in B cells and capable of diversified antibody production, but their output is tuned to barrier defence and tissue‐specific immune homeostasis.^14^ Accordingly, the immune phenotype observed in AH may reflect not simply defective maturation, but a shift towards short‐term, barrier‐oriented humoral activity at the expense of durable antigen‐specific memory. This may help explain why hypertrophic adenoids remain immunologically active and enlarged while appearing less efficiently organised for long‐term protective humoral imprinting.

At the ASC level, IgG and IgA subclass distributions were broadly comparable between groups, with IGHG1 remaining dominant; however, the AH group showed a higher fraction of IGHM‐positive ASCs. Rather than indicating a complete block in terminal differentiation, this pattern is more consistent with delayed or attenuated CSR. A plausible explanation is that chronic inflammatory microenvironmental cues uncouple proliferation from maturation in AH. Experimental studies have shown that hypoxia within germinal centres can alter B‐cell metabolism, limit AID expression and impair generation of high‐affinity, class‐switched antibody responses.^15^, ^16^ In the context of AH, where persistent antigen exposure and chronic upper‐airway inflammation are common, a similar microenvironmental constraint may contribute to expansion of GC and cycling B‐cell populations while limiting their qualitative maturation and export into the MBC pool. Taken together, these findings support a model in which AH is characterised not by absent humoral activation, but by sustained yet low‐efficiency humoral maturation.

### CD8 ^+^ TRM cells and the ‘double‐edged sword’ of mucosal defence

The immunological role of adenoids in paediatric health emerges as a double‐edged sword. While AH is the primary contributor to paediatric OSA and related morbidities, our data suggest that hypertrophy may reflect heightened immune activation in response to persistent antigen exposure. Although CD8^+^ TRM cell frequencies were comparable between groups, their clonotypic expansion and interferon‐stimulated transcriptional programmes were more prominent in AH. These highly active TRM cells function as primary ‘first responders’ at the nasopharyngeal ‘elbow,’ potentially reprogramming the epithelial niche towards an antiviral and antigen‐presentation programme.^17^ In addition to their potential role in frontline mucosal defence, activated CD8+ TRM cells may also amplify local inflammation through rapid production of IFN‐γ, TNF and chemotactic signals, thereby promoting epithelial activation and recruitment of additional immune cells.^18^ In the setting of persistent antigen exposure, such TRM‐driven ‘alarm’ responses may contribute to chronic immune stimulation rather than fully effective protection.

This active cellular defence complements the structural ‘barrier filter’ provided by the enlarged adenoid. The expansion of crypt epithelium in AH tissue substantially increases the antigen‐trapping surface area, facilitating the capture and presentation of inhaled pathogens—a process consistent with the robust TRM–epithelial interaction loop identified in our study. Simultaneously, the enlarged adenoid may contribute to local pathogen interception at the upper‐airway interface, although whether this translates into net protective benefit remains uncertain. Taken together, our findings suggest that AH is associated not only with airway obstruction but also with marked local immune remodelling at the upper‐airway mucosal interface. Whether this remodelling is ultimately protective, maladaptive or context dependent remains unresolved. These considerations may be relevant when weighing symptom relief against preservation of local immune tissue in surgical decision‐making.

### Limitations

Several limitations warrant consideration. First, the small sample size and single‐centre design may not fully capture the clinical heterogeneity of AH across diverse age groups. In addition, the absence of paired peripheral immune profiling limited our ability to fully distinguish local immune remodelling within adenoid tissue from broader systemic immune alterations. Second, potential cell‐capture biases inherent to scRNA‐seq could underrepresent rare cell populations. Third, the limited yield of epithelial cells necessitates that findings regarding epithelial remodelling and TRM–epithelial crosstalk be interpreted as exploratory. Finally, despite advanced repertoire profiling, the lack of robust in‐silico BCR‐specificity tools precluded definitive mapping of viral antigens to B‐cell expansion, a challenge that remains to be addressed by future functional studies.

## Conclusions

Adenoid hypertrophy is characterised by expanded yet qualitatively inefficient GC responses, marked by increased B‐cell proliferation but reduced SHM and CSR. CD8^+^ TRM in AH exhibit an activated antiviral transcriptional state. These findings suggest that AH is not merely an anatomic cause of upper‐airway obstruction but also an immunologically active mucosal state associated with ongoing immune remodelling and antiviral defence. Future studies with larger cohorts are needed to validate these immune and epithelial alterations, particularly in relation to the clinical outcomes of surgical interventions.

## Methods

### Participants and grouping

Participants were required to be aged 5–10 years and have OSA confirmed by baseline polysomnography (PSG), defined as an Obstructive Apnea–Hypopnea Index (OAHI) ≥ 1.^19^ Additionally, participants were required to have a tonsil size of ≥ grade II (Brodsky) to be considered candidates for AT. The study excluded children with primary comorbid conditions, such as craniofacial anomalies, cerebral palsy, cardiac arrhythmia and developmental delay. All children underwent preoperative adenoid evaluation using flexible nasopharyngoscopy (Figure 1b) and were stratified into the AH group (Grade III–IV) and the control group (Grade I–II).^20^ The term ‘control’ is used in a clinical–anatomical sense to denote adenoids within the non‐hypertrophic size range under a shared surgical context, rather than to represent immunologically healthy tissue.

### Enrolment and ethics

Participants were enrolled between July 2024 and April 2025 at BenQ Medical Center, the Fourth School of Clinical Medicine, Nanjing Medical University. All the participants provided written informed consent and the research protocol was approved by the research ethics board at the BenQ Medical Center (2024‐KL014).

### Questionnaire assessment

Symptom burden was assessed using the Sleep Related Breathing Disorder Scale of the Pediatric Sleep Questionnaire (PSQ), a 22‐item standardised survey ranging from 0 to 1.^21^ Quality of life was assessed using the Obstructive Sleep Apnea‐18 (OSA‐18), an 18‐item questionnaire, with higher scores indicating worse quality of life.^22^

### Sample collection and processing

Adenoid tissues were obtained at the time of adenoidectomy. Samples were immediately placed in cold sterile buffer and transported on ice for prompt downstream single‐cell processing.

### Single‐cell immune repertoire and transcriptome analysis

Fresh adenoid tissue was enzymatically and mechanically dissociated into single‐cell suspensions, and cell viability was assessed with trypan blue. Viable cells were loaded onto the 10× Genomics Chromium platform to generate 5′ scRNA‐seq libraries, enabling concurrent whole‐transcriptome and full‐length V(D)J capture. Following reverse transcription and amplification, gene‐expression and V(D)J libraries were sequenced on a high‐throughput platform. Raw data were processed with Cell Ranger for demultiplexing, alignment and feature counting.

### Immunofluorescence staining

Formalin‐fixed, paraffin‐embedded (FFPE) adenoid tissue sections were obtained from 8 children (AH, *n* = 4; control, *n* = 4). Sections were deparaffinised, rehydrated and subjected to heat‐induced antigen retrieval. After blocking, a sequential staining protocol was performed to allow the use of multiple antibodies from the same species. For proliferating B cells, sections were incubated overnight at 4°C with primary antibodies against CD19 (rabbit, 27949‐1‐AP, 1:1000; Proteintech, Wuhan) and Ki67 (mouse, YM‐B20013, 1:800; Youmeng Biotechnology, Nanjing). For validation of CD8+ TRM cells, additional sections were stained with antibodies against CD8 (rabbit, YM‐M02295, 1:2000; Youmeng Biotechnology, Nanjing), CD69 (rabbit, YM‐B22777, 1:1400; Youmeng Biotechnology, Nanjing) and CD103 (rabbit, ab224202, 1:5000; Abcam). Between each staining cycle, the previously bound antibodies were removed by heat‐induced treatment to prevent cross‐reactivity. Nuclei were counterstained with DAPI, and representative images were acquired using a fluorescence microscope.

### Statistical analysis

Data were analysed using R software (version 4.4.2), supplemented by GraphPad Prism (version 9) for graphical visualisation. Continuous variables are presented as medians [interquartile ranges (IQR)], and categorical variables as frequencies and percentages. Median differences and 95% confidence intervals (CIs) were estimated using the Hodges–Lehmann method for non‐normally distributed variables. Associations between group and categorical variables were assessed using odds ratios (ORs) with 95% CIs.

For single‐cell transcriptomic data, dimensionality reduction [principal component analysis and uniform manifold approximation and projection (UMAP)], clustering and differential gene‐expression analyses were performed using the Seurat package (version 5.2.0).^23^ To reduce potential technical variation across samples, scRNA‐seq datasets were integrated using the canonical correlation analysis (CCA)‐based integration workflow implemented in Seurat before downstream dimensionality reduction and clustering. Differential expression was evaluated using the Wilcoxon rank sum test, with *P*‐values adjusted for multiple comparisons using the Benjamini–Hochberg false discovery rate (FDR) method. Gene Set Enrichment Analysis (GSEA): To explore the functional states of B‐cell subsets, GSEA was performed using the MSigDB HALLMARK and C7 (immunologic signatures) collections. A permutation test with 1000 iterations was used to identify significantly enriched pathways. Results were considered significant with a Normalized Enrichment Score (NES) > 1.0 and FDR < 0.05.

Raw BCR and TCR V(D)J sequencing data were initially processed using the Cell Ranger (10× Genomics) pipeline for demultiplexing, read alignment, V(D)J assembly and preliminary clonotype assignment. Germline V, D and J gene segment annotation, CDR3 sequence extraction and isotype calling were performed using IMGT reference databases. Basic repertoire features, including V and J gene usage frequencies, V–J pairing patterns, CDR3 length distributions and clonal expansion profiles, were first examined to characterise global repertoire architecture.

BCR repertoire analyses were performed using the Immcantation framework, including the Change‐O, scoper and SHazaM packages, to conduct clonal assignment, class‐switch recombination (CSR) and somatic hypermutation (SHM) analyses.^24^ TCR clonotypes were identified based on V(D)J rearrangements, and antigen specificity was annotated using the VDJdb database, which contains experimentally validated TCR–epitope interactions across multiple viral pathogens. All statistical tests were two‐sided, and *P* < 0.05 was considered statistically significant unless otherwise specified.

Immunofluorescence images were analysed using the QuPath software (version 0.5.1).^25^ Germinal‐centre (GC) regions were manually annotated on each section, and cell detection was performed within the selected GC regions. Nuclei were segmented using the DAPI channel, and cells were classified by marker intensity; Ki67 positivity was defined by nuclear Ki67 signal, and CD19 positivity by whole‐cell or cytoplasmic CD19 signal. For each case, all GC annotations from the same section were pooled, and the proportion of CD19^+^Ki67^+^ cells among CD19^+^ cells was used as the primary quantitative measure.

## Author contributions

**Yuanyuan Lu:** Writing – review and editing; conceptualization; supervision; resources. **Jing Li:** Methodology; validation. **Xiao Han:** Visualization. **Zhenkun Yu:** Writing – review and editing; funding acquisition; supervision; resources; project administration. **Yufei Pan:** Methodology; validation. **Chao Wang:** Conceptualization; methodology; investigation; data curation; formal analysis; writing – original draft. **Kai Sun:** Validation; investigation.

## Conflict of interest

The authors declare no conflict of interest.

## Ethics approval

The research protocol was approved by the research ethics board at the BenQ Medical Center (2024‐KL014).

## Consent to participate

Written informed consent was obtained from the parents or legal guardians of all participants included in the study.

## Supporting information


Supplementary figure 1

Supplementary table 1

Supplementary table 2

## Data Availability

All data generated for this manuscript have been included in this article. The single‐cell sequencing and repertoire data supporting the findings of this study are available from the corresponding author upon reasonable request.
