# Supplementary figures and images for: Single‐cell and repertoire profiling reveals immune remodelling in paediatric upper airway: insights from adenoid hypertrophy
Source: Clin Transl Immunology. 2026 Jun 5;15(6):e70101. doi: 10.1002/cti2.70101 (PMC13238750; doi:10.1002/cti2.70101)

A

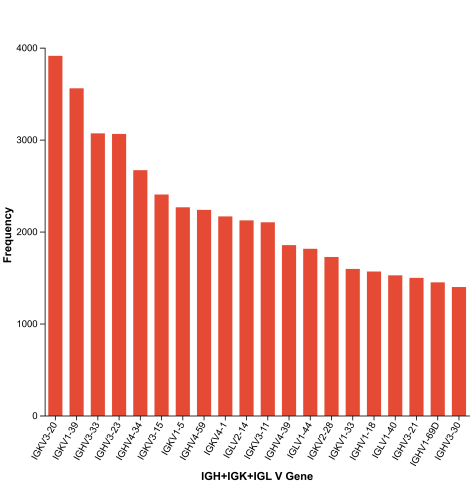

B

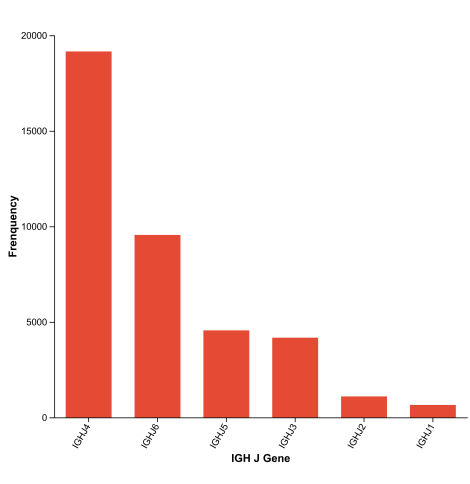

C

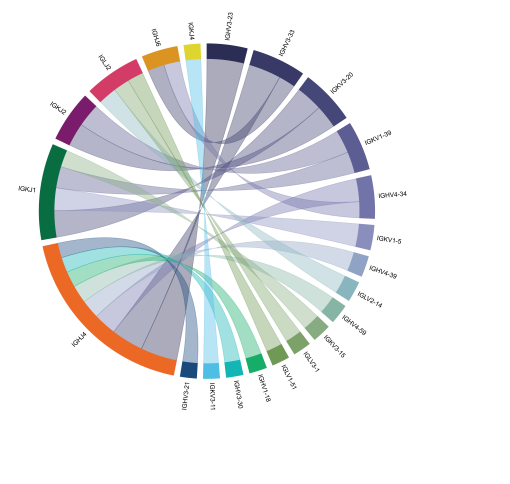

D

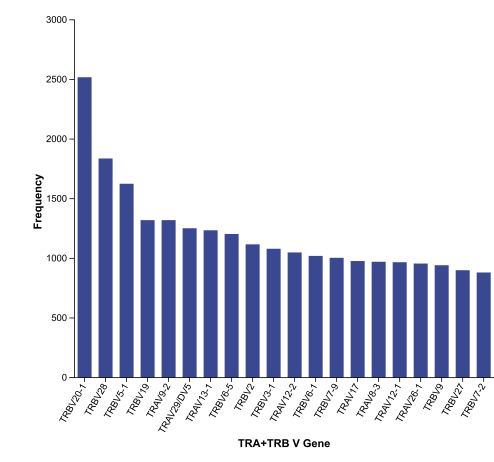

E

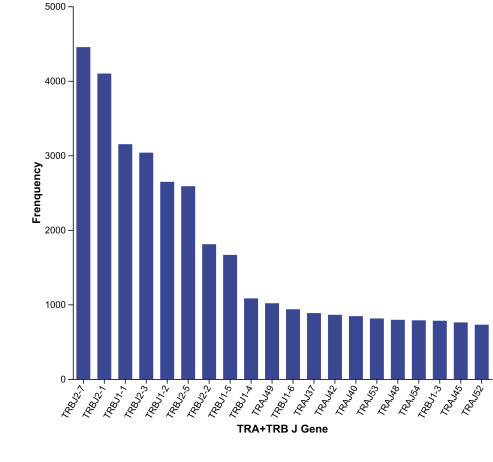

F

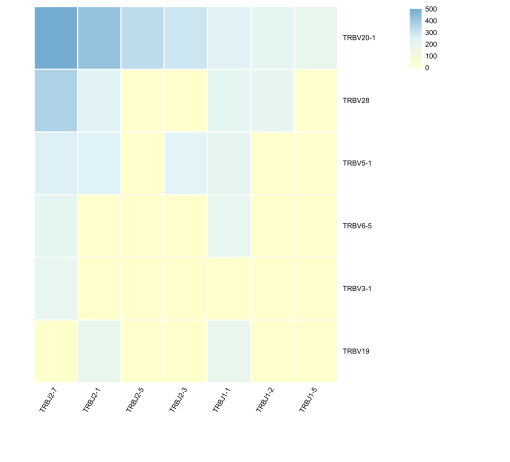

G

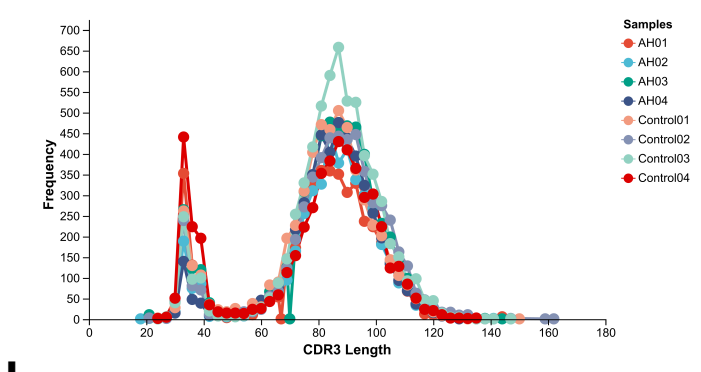

H

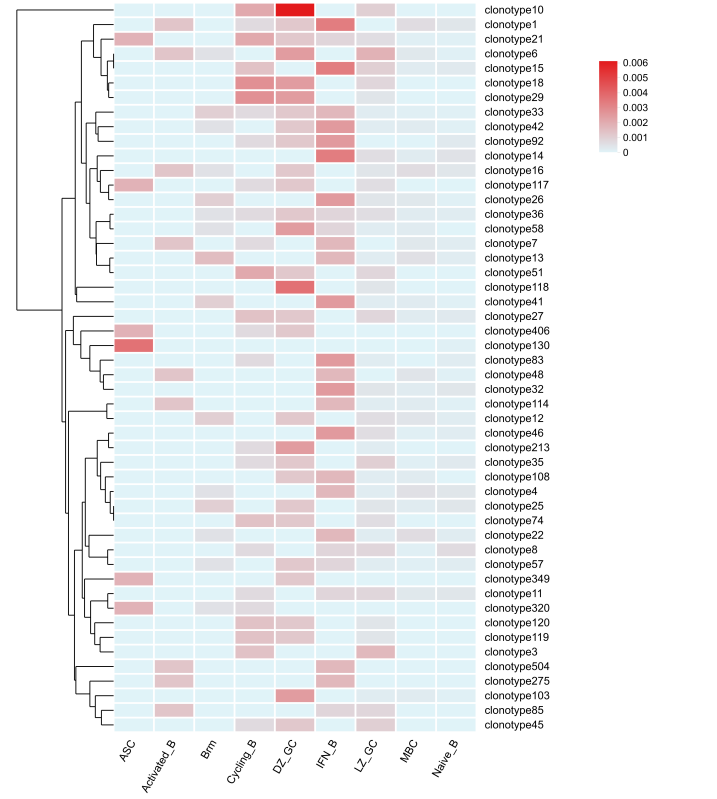

I

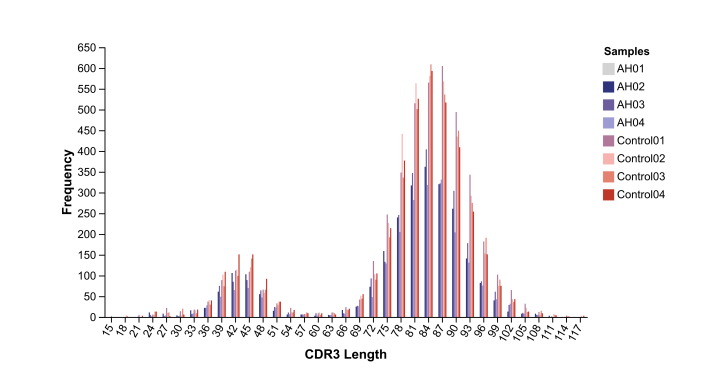

Supplement: Supplementary file 1 — Supplementary figure 1 [file CTI2-15-e70101-s001.pdf]
